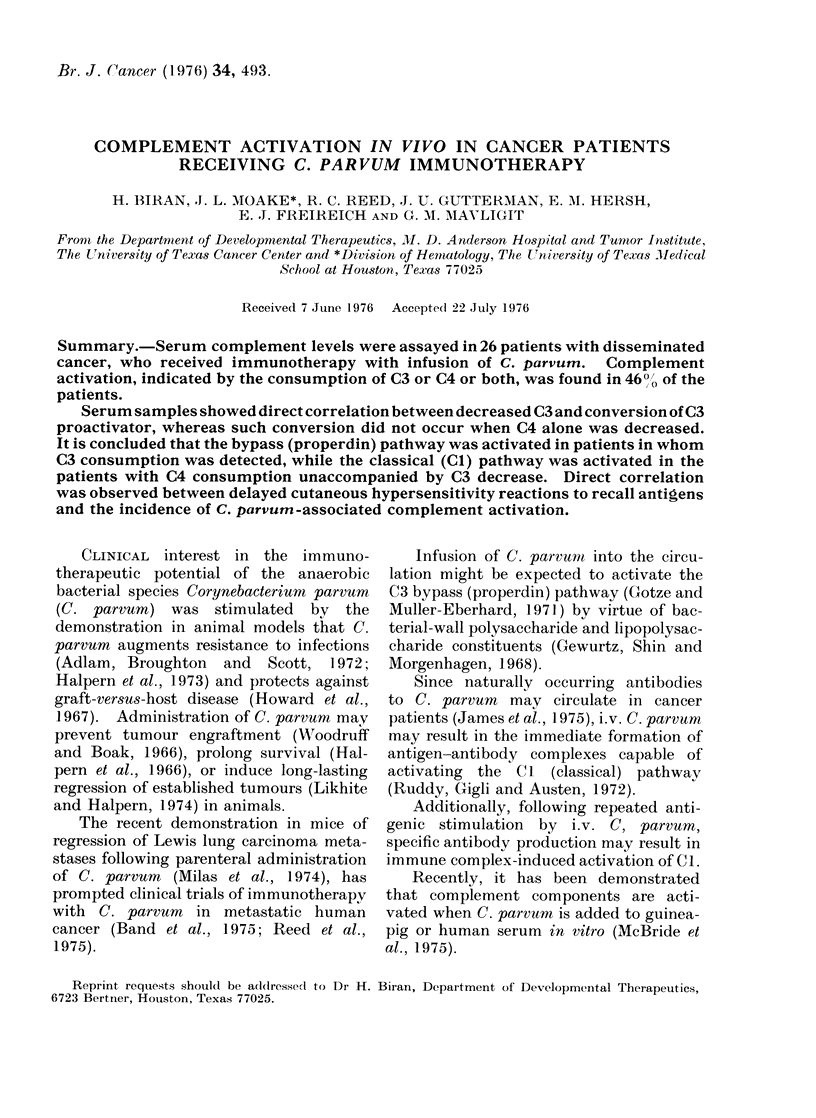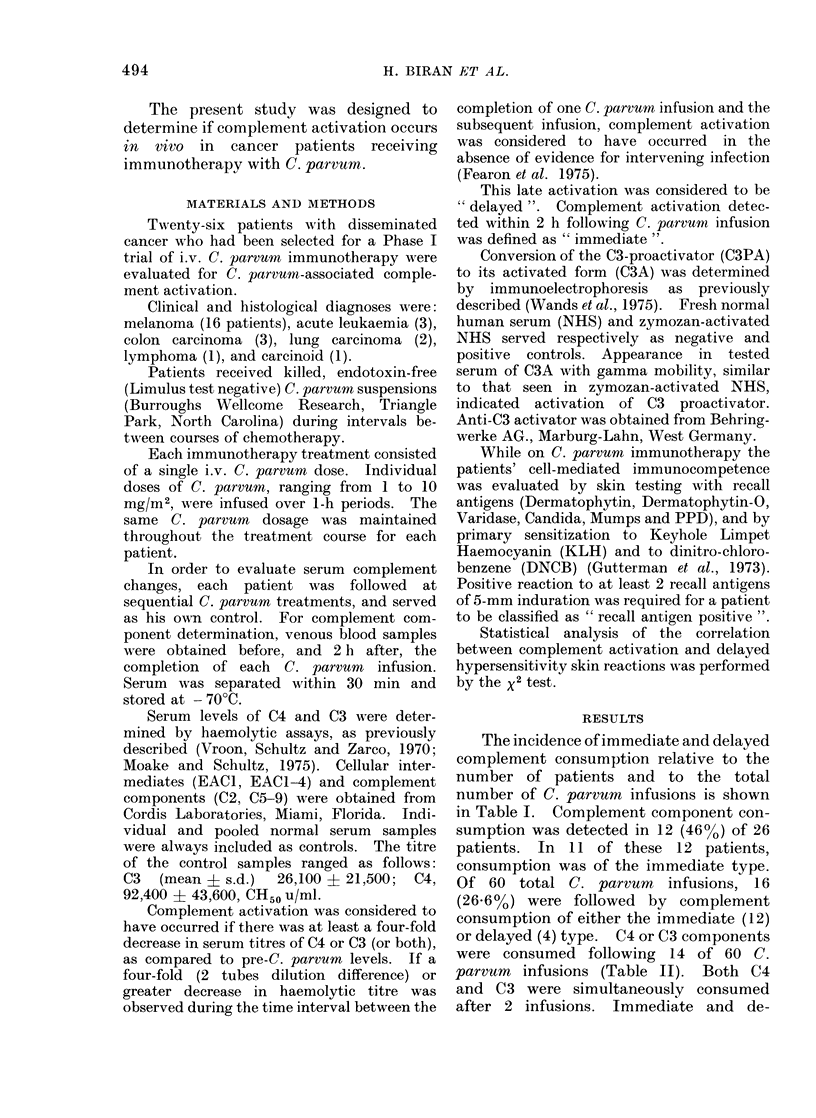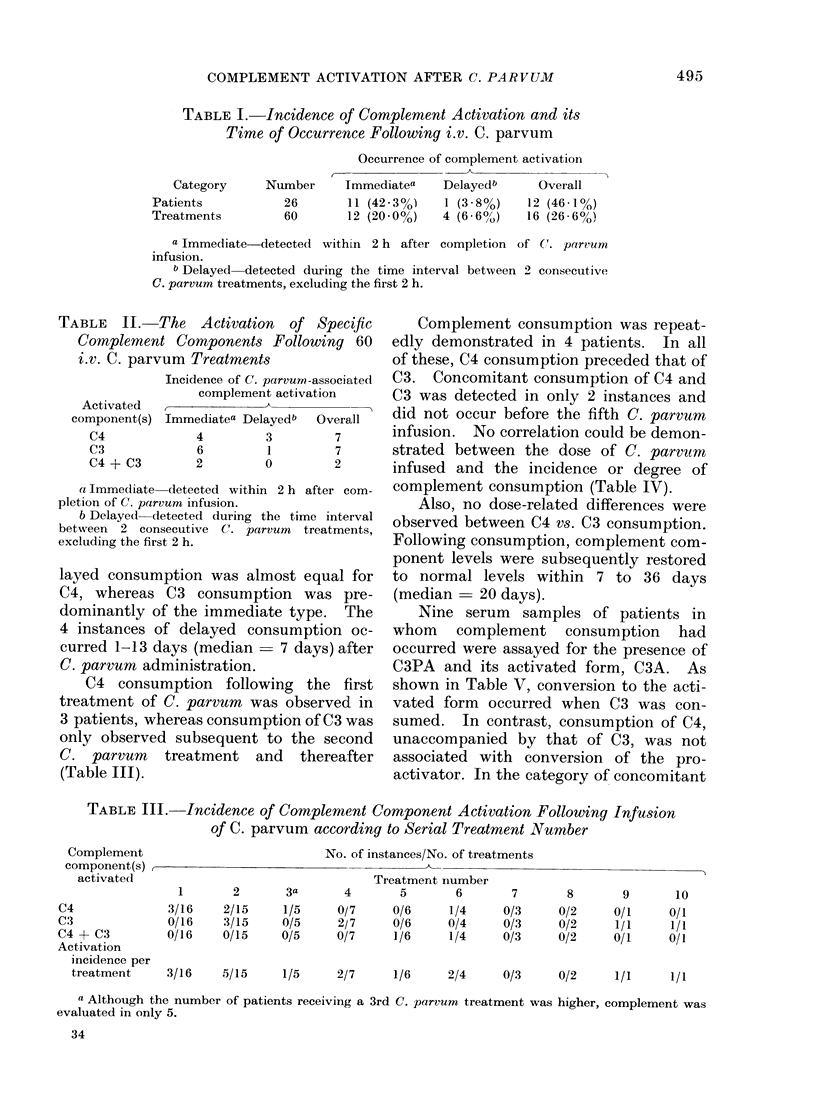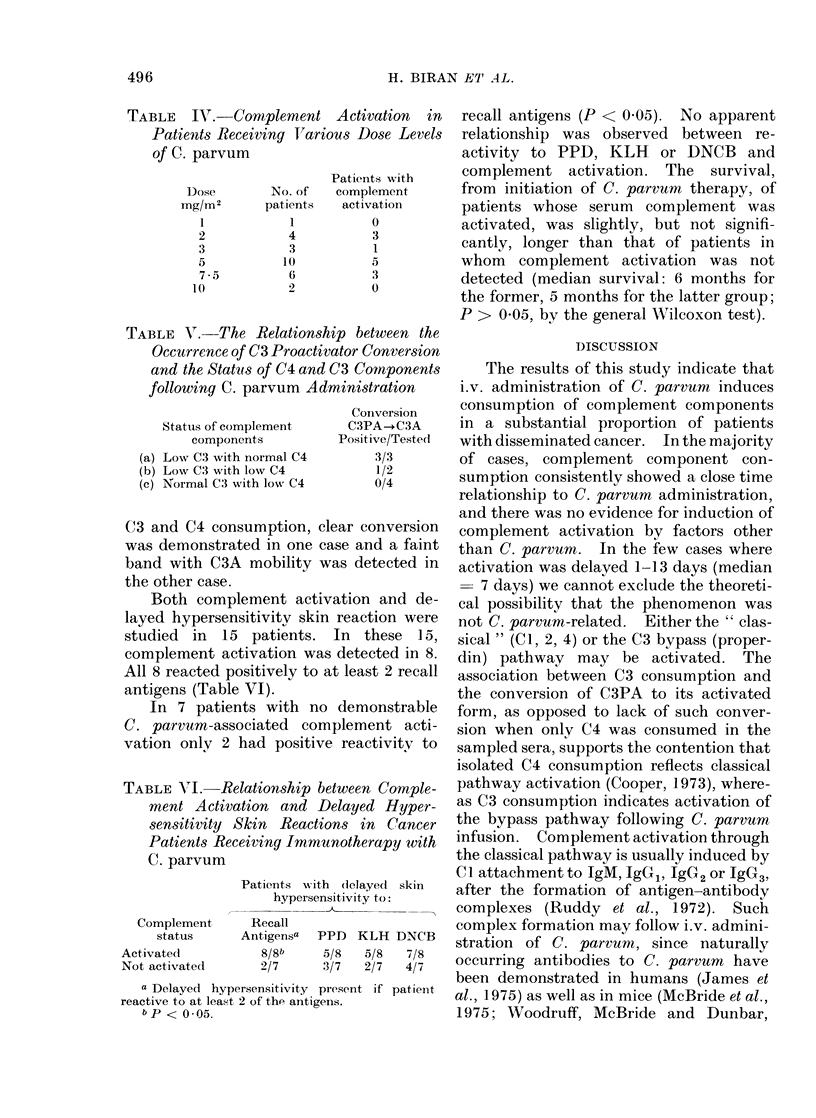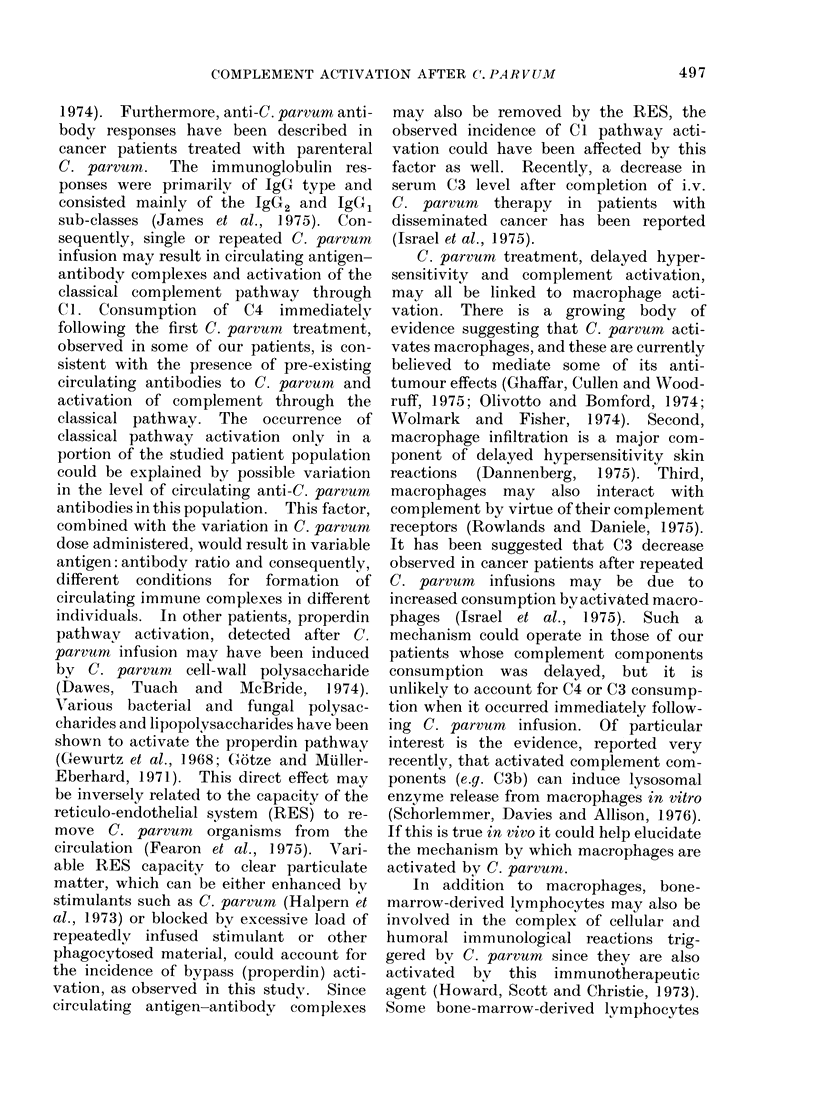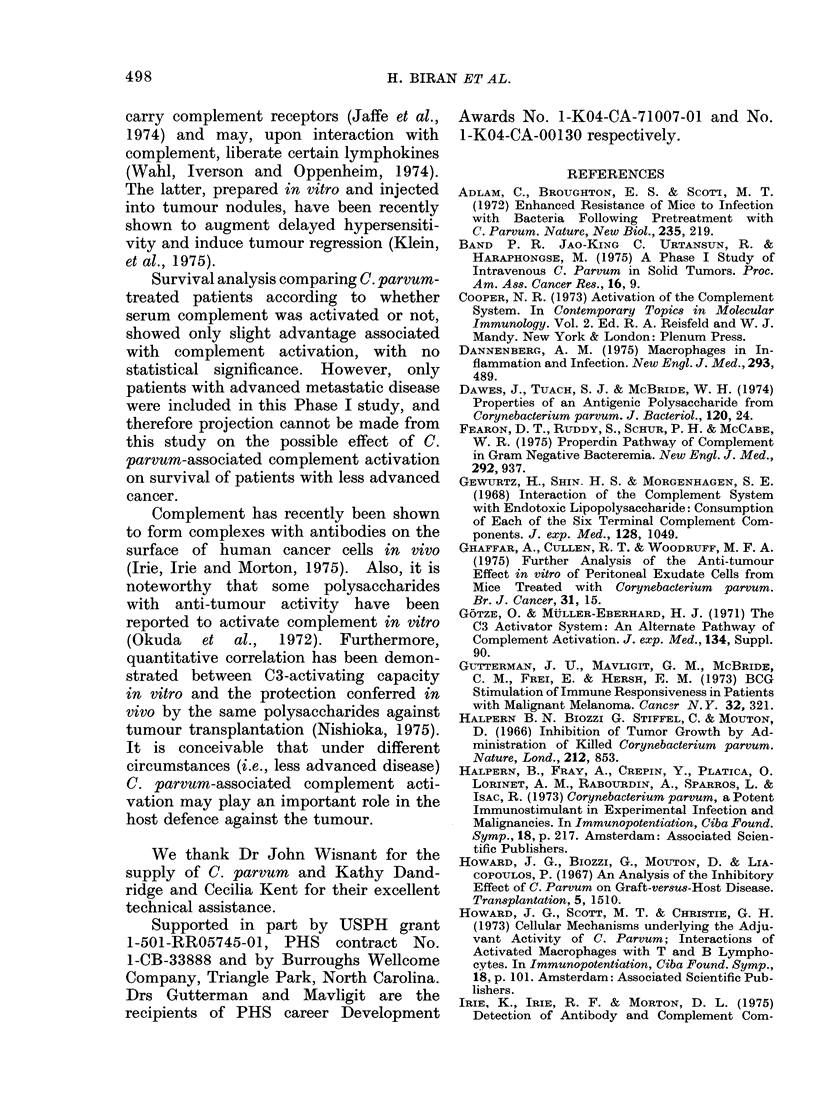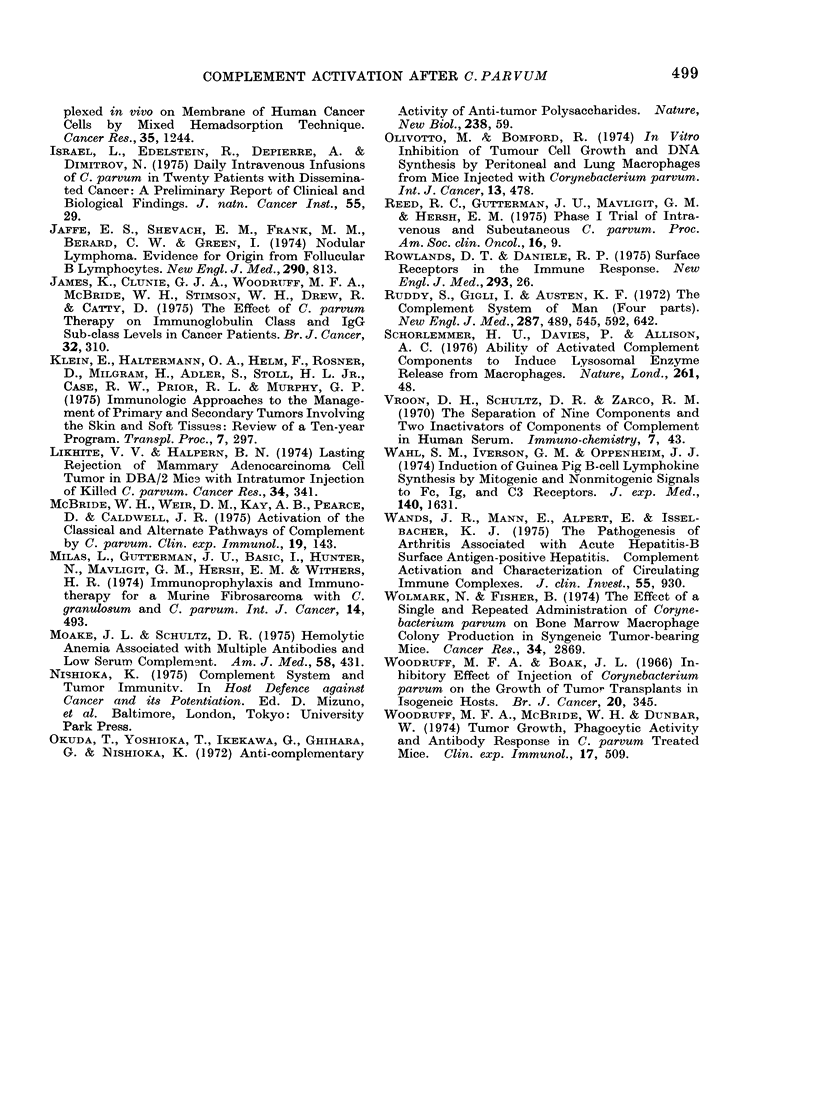# Complement activation in vivo in cancer patients receiving C. parvum immunotherapy.

**DOI:** 10.1038/bjc.1976.203

**Published:** 1976-11

**Authors:** H. Biran, J. L. Moake, R. C. Reed, J. U. Gutterman, E. M. Hersh, E. J. Freireich, G. M. Mavligit

## Abstract

Serum complement levels were assayed in 26 patients with disseminated cancer, who received immunotherapy with infusion of C. parvum. Complement activation, indicated by the consumption of C3 or C4 or both, was found in 46% of the patients. Serum samples showed direct correlation between decreased C3 and conversion of C3 proactivator, whereas such conversion did not occur when C4 alone was decreased. It is concluded that the bypass (properdin) pathway was activated in patients in whom C3 consumption was detected, while the classical (C1) pathway was activated in the patients with C4 consumption unaccompanied by C3 decrease. Direct correlation was observed between delayed cutaneous hypersensitivity reactions to recall antigens and the incidence of C. parvum-associated complement activation.


Br. J. Cancer (1976) 34, 493.

### COMPLEMENT ACTIVATION *IN VIVO* IN CANCER PATIENTS RECEIVING *C. PARVUM* IMMUNOTHERAPY

H. BIRAN, J. L. MOAKE*, R. C. REED, J. U. GUTTERMAN, E. M. HERSH, E. J. FREIREICH AND G. M. MAVLIGIT

*From the Department of Developmental Therapeutics, M. D. Anderson Hospital and Tumor Institute, The University of Texas Cancer Center and *Division of Hematology, The University of Texas Medical School at Houston, Texas 77025*

Received 7 June 1976 Accepted 22 July 1976

**Summary.—Serum complement levels were assayed in 26 patients with disseminated cancer, who received immunotherapy with infusion of *C. parvum*. Complement activation, indicated by the consumption of C3 or C4 or both, was found in 46% of the patients.**

**Serum samples showed direct correlation between decreased C3 and conversion of C3 proactivator, whereas such conversion did not occur when C4 alone was decreased. It is concluded that the bypass (properdin) pathway was activated in patients in whom C3 consumption was detected, while the classical (C1) pathway was activated in the patients with C4 consumption unaccompanied by C3 decrease. Direct correlation was observed between delayed cutaneous hypersensitivity reactions to recall antigens and the incidence of *C. parvum*-associated complement activation.**

CLINICAL interest in the immunotherapeutic potential of the anaerobic bacterial species *Corynebacterium parvum* (*C. parvum*) was stimulated by the demonstration in animal models that *C. parvum* augments resistance to infections (Adlam, Broughton and Scott, 1972; Halpern *et al.*, 1973) and protects against graft-*versus*-host disease (Howard *et al.*, 1967). Administration of *C. parvum* may prevent tumour engraftment (Woodruff and Boak, 1966), prolong survival (Halpern *et al.*, 1966), or induce long-lasting regression of established tumours (Likhite and Halpern, 1974) in animals.

The recent demonstration in mice of regression of Lewis lung carcinoma metastases following parenteral administration of *C. parvum* (Milas *et al.*, 1974), has prompted clinical trials of immunotherapy with *C. parvum* in metastatic human cancer (Band *et al.*, 1975; Reed *et al.*, 1975).

Infusion of *C. parvum* into the circulation might be expected to activate the C3 bypass (properdin) pathway (Gotze and Muller-Eberhard, 1971) by virtue of bacterial-wall polysaccharide and lipopolysaccharide constituents (Gewurtz, Shin and Morgenhagen, 1968).

Since naturally occurring antibodies to *C. parvum* may circulate in cancer patients (James *et al.*, 1975), i.v. *C. parvum* may result in the immediate formation of antigen–antibody complexes capable of activating the C1 (classical) pathway (Ruddy, Gigli and Austen, 1972).

Additionally, following repeated antigenic stimulation by i.v. *C, parvum*, specific antibody production may result in immune complex-induced activation of C1.

Recently, it has been demonstrated that complement components are activated when *C. parvum* is added to guinea-pig or human serum *in vitro* (McBride *et al.*, 1975).

Reprint requests should be addressed to Dr H. Biran, Department of Developmental Therapeutics, 6723 Bertner, Houston, Texas 77025.

The present study was designed to determine if complement activation occurs *in vivo* in cancer patients receiving immunotherapy with *C. parvum.*

### MATERIALS AND METHODS

Twenty-six patients with disseminated cancer who had been selected for a Phase I trial of i.v. *C. parvum* immunotherapy were evaluated for *C. parvum*-associated complement activation.

Clinical and histological diagnoses were: melanoma (16 patients), acute leukaemia (3), colon carcinoma (3), lung carcinoma (2), lymphoma (1), and carcinoid (1).

Patients received killed, endotoxin-free (Limulus test negative) *C. parvum* suspensions (Burroughs Wellcome Research, Triangle Park, North Carolina) during intervals between courses of chemotherapy.

Each immunotherapy treatment consisted of a single i.v. *C. parvum* dose. Individual doses of *C. parvum*, ranging from 1 to 10 mg/m$^2$, were infused over 1-h periods. The same *C. parvum* dosage was maintained throughout the treatment course for each patient.

In order to evaluate serum complement changes, each patient was followed at sequential *C. parvum* treatments, and served as his own control. For complement component determination, venous blood samples were obtained before, and 2 h after, the completion of each *C. parvum* infusion. Serum was separated within 30 min and stored at $-70$°C.

Serum levels of C4 and C3 were determined by haemolytic assays, as previously described (Vroon, Schultz and Zarco, 1970; Moake and Schultz, 1975). Cellular intermediates (EAC1, EAC1–4) and complement components (C2, C5–9) were obtained from Cordis Laboratories, Miami, Florida. Individual and pooled normal serum samples were always included as controls. The titre of the control samples ranged as follows: C3 (mean $\pm$ s.d.) 26,100 $\pm$ 21,500; C4, 92,400 $\pm$ 43,600, $CH_{50}$ u/ml.

Complement activation was considered to have occurred if there was at least a four-fold decrease in serum titres of C4 or C3 (or both), as compared to pre-*C. parvum* levels. If a four-fold (2 tubes dilution difference) or greater decrease in haemolytic titre was observed during the time interval between the completion of one *C. parvum* infusion and the subsequent infusion, complement activation was considered to have occurred in the absence of evidence for intervening infection (Fearon *et al.* 1975).

This late activation was considered to be " delayed ". Complement activation detected within 2 h following *C. parvum* infusion was defined as " immediate ".

Conversion of the C3-proactivator (C3PA) to its activated form (C3A) was determined by immunoelectrophoresis as previously described (Wands *et al.*, 1975). Fresh normal human serum (NHS) and zymozan-activated NHS served respectively as negative and positive controls. Appearance in tested serum of C3A with gamma mobility, similar to that seen in zymozan-activated NHS, indicated activation of C3 proactivator. Anti-C3 activator was obtained from Behringwerke AG., Marburg-Lahn, West Germany.

While on *C. parvum* immunotherapy the patients' cell-mediated immunocompetence was evaluated by skin testing with recall antigens (Dermatophytin, Dermatophytin-O, Varidase, Candida, Mumps and PPD), and by primary sensitization to Keyhole Limpet Haemocyanin (KLH) and to dinitro-chlorobenzene (DNCB) (Gutterman *et al.*, 1973). Positive reaction to at least 2 recall antigens of 5-mm induration was required for a patient to be classified as " recall antigen positive ".

Statistical analysis of the correlation between complement activation and delayed hypersensitivity skin reactions was performed by the $\chi^2$ test.

### RESULTS

The incidence of immediate and delayed complement consumption relative to the number of patients and to the total number of *C. parvum* infusions is shown in Table I. Complement component consumption was detected in 12 (46%) of 26 patients. In 11 of these 12 patients, consumption was of the immediate type. Of 60 total *C. parvum* infusions, 16 (26·6%) were followed by complement consumption of either the immediate (12) or delayed (4) type. C4 or C3 components were consumed following 14 of 60 *C. parvum* infusions (Table II). Both C4 and C3 were simultaneously consumed after 2 infusions. Immediate and de-

TABLE I.—*Incidence of Complement Activation and its Time of Occurrence Following i.v.* C. parvum

| Category | Number | Occurrence of complement activation | | |
|---|---|---|---|---|
| | | Immediate[a] | Delayed[b] | Overall |
| Patients | 26 | 11 (42·3%) | 1 (3·8%) | 12 (46·1%) |
| Treatments | 60 | 12 (20·0%) | 4 (6·6%) | 16 (26·6%) |

[a] Immediate—detected within 2 h after completion of *C. parvum* infusion.

[b] Delayed—detected during the time interval between 2 consecutive *C. parvum* treatments, excluding the first 2 h.

TABLE II.—*The Activation of Specific Complement Components Following* 60 *i.v.* C. parvum *Treatments*

| Activated component(s) | Incidence of *C. parvum*-associated complement activation | | |
|---|---|---|---|
| | Immediate[a] | Delayed[b] | Overall |
| C4 | 4 | 3 | 7 |
| C3 | 6 | 1 | 7 |
| C4 + C3 | 2 | 0 | 2 |

*a* Immediate—detected within 2 h after completion of *C. parvum* infusion.

*b* Delayed—detected during the time interval between 2 consecutive *C. parvum* treatments, excluding the first 2 h.

layed consumption was almost equal for C4, whereas C3 consumption was predominantly of the immediate type. The 4 instances of delayed consumption occurred 1–13 days (median = 7 days) after *C. parvum* administration.

C4 consumption following the first treatment of *C. parvum* was observed in 3 patients, whereas consumption of C3 was only observed subsequent to the second *C. parvum* treatment and thereafter (Table III).

Complement consumption was repeatedly demonstrated in 4 patients. In all of these, C4 consumption preceded that of C3. Concomitant consumption of C4 and C3 was detected in only 2 instances and did not occur before the fifth *C. parvum* infusion. No correlation could be demonstrated between the dose of *C. parvum* infused and the incidence or degree of complement consumption (Table IV).

Also, no dose-related differences were observed between C4 *vs.* C3 consumption. Following consumption, complement component levels were subsequently restored to normal levels within 7 to 36 days (median = 20 days).

Nine serum samples of patients in whom complement consumption had occurred were assayed for the presence of C3PA and its activated form, C3A. As shown in Table V, conversion to the activated form occurred when C3 was consumed. In contrast, consumption of C4, unaccompanied by that of C3, was not associated with conversion of the proactivator. In the category of concomitant

TABLE III.—*Incidence of Complement Component Activation Following Infusion of* C. parvum *according to Serial Treatment Number*

| Complement component(s) activated | No. of instances/No. of treatments | | | | | | | | | |
|---|---|---|---|---|---|---|---|---|---|---|
| | Treatment number | | | | | | | | | |
| | 1 | 2 | 3[a] | 4 | 5 | 6 | 7 | 8 | 9 | 10 |
| C4 | 3/16 | 2/15 | 1/5 | 0/7 | 0/6 | 1/4 | 0/3 | 0/2 | 0/1 | 0/1 |
| C3 | 0/16 | 3/15 | 0/5 | 2/7 | 0/6 | 0/4 | 0/3 | 0/2 | 1/1 | 1/1 |
| C4 + C3 | 0/16 | 0/15 | 0/5 | 0/7 | 1/6 | 1/4 | 0/3 | 0/2 | 0/1 | 0/1 |
| Activation incidence per treatment | 3/16 | 5/15 | 1/5 | 2/7 | 1/6 | 2/4 | 0/3 | 0/2 | 1/1 | 1/1 |

[a] Although the number of patients receiving a 3rd *C. parvum* treatment was higher, complement was evaluated in only 5.

TABLE IV.—*Complement Activation in Patients Receiving Various Dose Levels of* C. parvum

| Dose mg/m² | No. of patients | Patients with complement activation |
|---|---|---|
| 1 | 1 | 0 |
| 2 | 4 | 3 |
| 3 | 3 | 1 |
| 5 | 10 | 5 |
| 7·5 | 6 | 3 |
| 10 | 2 | 0 |

TABLE V.—*The Relationship between the Occurrence of C3 Proactivator Conversion and the Status of C4 and C3 Components following* C. parvum *Administration*

| Status of complement components | Conversion C3PA→C3A Positive/Tested |
|---|---|
| (a) Low C3 with normal C4 | 3/3 |
| (b) Low C3 with low C4 | 1/2 |
| (c) Normal C3 with low C4 | 0/4 |

C3 and C4 consumption, clear conversion was demonstrated in one case and a faint band with C3A mobility was detected in the other case.

Both complement activation and delayed hypersensitivity skin reaction were studied in 15 patients. In these 15, complement activation was detected in 8. All 8 reacted positively to at least 2 recall antigens (Table VI).

In 7 patients with no demonstrable *C. parvum*-associated complement activation only 2 had positive reactivity to recall antigens ($P < 0{\cdot}05$). No apparent relationship was observed between reactivity to PPD, KLH or DNCB and complement activation. The survival, from initiation of *C. parvum* therapy, of patients whose serum complement was activated, was slightly, but not significantly, longer than that of patients in whom complement activation was not detected (median survival: 6 months for the former, 5 months for the latter group; $P > 0{\cdot}05$, by the general Wilcoxon test).

TABLE VI.—*Relationship between Complement Activation and Delayed Hypersensitivity Skin Reactions in Cancer Patients Receiving Immunotherapy with* C. parvum

| Complement status | Patients with delayed skin hypersensitivity to: Recall Antigens[a] | PPD | KLH | DNCB |
|---|---|---|---|---|
| Activated | 8/8[b] | 5/8 | 5/8 | 7/8 |
| Not activated | 2/7 | 3/7 | 2/7 | 4/7 |

[a] Delayed hypersensitivity present if patient reactive to at least 2 of the antigens.

[b] $P < 0{\cdot}05$.

### DISCUSSION

The results of this study indicate that i.v. administration of *C. parvum* induces consumption of complement components in a substantial proportion of patients with disseminated cancer. In the majority of cases, complement component consumption consistently showed a close time relationship to *C. parvum* administration, and there was no evidence for induction of complement activation by factors other than *C. parvum.* In the few cases where activation was delayed 1–13 days (median = 7 days) we cannot exclude the theoretical possibility that the phenomenon was not *C. parvum*-related. Either the " classical " (C1, 2, 4) or the C3 bypass (properdin) pathway may be activated. The association between C3 consumption and the conversion of C3PA to its activated form, as opposed to lack of such conversion when only C4 was consumed in the sampled sera, supports the contention that isolated C4 consumption reflects classical pathway activation (Cooper, 1973), whereas C3 consumption indicates activation of the bypass pathway following *C. parvum* infusion. Complement activation through the classical pathway is usually induced by C1 attachment to IgM, $IgG_1$, $IgG_2$ or $IgG_3$, after the formation of antigen–antibody complexes (Ruddy *et al.*, 1972). Such complex formation may follow i.v. administration of *C. parvum*, since naturally occurring antibodies to *C. parvum* have been demonstrated in humans (James *et al.*, 1975) as well as in mice (McBride *et al.*, 1975; Woodruff, McBride and Dunbar,

1974). Furthermore, anti-*C. parvum* antibody responses have been described in cancer patients treated with parenteral *C. parvum*. The immunoglobulin responses were primarily of IgG type and consisted mainly of the $IgG_2$ and $IgG_1$ sub-classes (James *et al.*, 1975). Consequently, single or repeated *C. parvum* infusion may result in circulating antigen–antibody complexes and activation of the classical complement pathway through C1. Consumption of C4 immediately following the first *C. parvum* treatment, observed in some of our patients, is consistent with the presence of pre-existing circulating antibodies to *C. parvum* and activation of complement through the classical pathway. The occurrence of classical pathway activation only in a portion of the studied patient population could be explained by possible variation in the level of circulating anti-*C. parvum* antibodies in this population. This factor, combined with the variation in *C. parvum* dose administered, would result in variable antigen: antibody ratio and consequently, different conditions for formation of circulating immune complexes in different individuals. In other patients, properdin pathway activation, detected after *C. parvum* infusion may have been induced by *C. parvum* cell-wall polysaccharide (Dawes, Tuach and McBride, 1974). Various bacterial and fungal polysaccharides and lipopolysaccharides have been shown to activate the properdin pathway (Gewurtz *et al.*, 1968; Götze and Müller-Eberhard, 1971). This direct effect may be inversely related to the capacity of the reticulo-endothelial system (RES) to remove *C. parvum* organisms from the circulation (Fearon *et al.*, 1975). Variable RES capacity to clear particulate matter, which can be either enhanced by stimulants such as *C. parvum* (Halpern *et al.*, 1973) or blocked by excessive load of repeatedly infused stimulant or other phagocytosed material, could account for the incidence of bypass (properdin) activation, as observed in this study. Since circulating antigen–antibody complexes may also be removed by the RES, the observed incidence of C1 pathway activation could have been affected by this factor as well. Recently, a decrease in serum C3 level after completion of i.v. *C. parvum* therapy in patients with disseminated cancer has been reported (Israel *et al.*, 1975).

*C. parvum* treatment, delayed hypersensitivity and complement activation, may all be linked to macrophage activation. There is a growing body of evidence suggesting that *C. parvum* activates macrophages, and these are currently believed to mediate some of its anti-tumour effects (Ghaffar, Cullen and Woodruff, 1975; Olivotto and Bomford, 1974; Wolmark and Fisher, 1974). Second, macrophage infiltration is a major component of delayed hypersensitivity skin reactions (Dannenberg, 1975). Third, macrophages may also interact with complement by virtue of their complement receptors (Rowlands and Daniele, 1975). It has been suggested that C3 decrease observed in cancer patients after repeated *C. parvum* infusions may be due to increased consumption by activated macrophages (Israel *et al.*, 1975). Such a mechanism could operate in those of our patients whose complement components consumption was delayed, but it is unlikely to account for C4 or C3 consumption when it occurred immediately following *C. parvum* infusion. Of particular interest is the evidence, reported very recently, that activated complement components (*e.g.* C3b) can induce lysosomal enzyme release from macrophages *in vitro* (Schorlemmer, Davies and Allison, 1976). If this is true *in vivo* it could help elucidate the mechanism by which macrophages are activated by *C. parvum*.

In addition to macrophages, bone-marrow-derived lymphocytes may also be involved in the complex of cellular and humoral immunological reactions triggered by *C. parvum* since they are also activated by this immunotherapeutic agent (Howard, Scott and Christie, 1973). Some bone-marrow-derived lymphocytes carry complement receptors (Jaffe *et al.*, 1974) and may, upon interaction with complement, liberate certain lymphokines (Wahl, Iverson and Oppenheim, 1974). The latter, prepared *in vitro* and injected into tumour nodules, have been recently shown to augment delayed hypersensitivity and induce tumour regression (Klein, *et al.*, 1975).

Survival analysis comparing *C. parvum*-treated patients according to whether serum complement was activated or not, showed only slight advantage associated with complement activation, with no statistical significance. However, only patients with advanced metastatic disease were included in this Phase I study, and therefore projection cannot be made from this study on the possible effect of *C. parvum*-associated complement activation on survival of patients with less advanced cancer.

Complement has recently been shown to form complexes with antibodies on the surface of human cancer cells *in vivo* (Irie, Irie and Morton, 1975). Also, it is noteworthy that some polysaccharides with anti-tumour activity have been reported to activate complement *in vitro* (Okuda *et al.*, 1972). Furthermore, quantitative correlation has been demonstrated between C3-activating capacity *in vitro* and the protection conferred *in vivo* by the same polysaccharides against tumour transplantation (Nishioka, 1975). It is conceivable that under different circumstances (*i.e.*, less advanced disease) *C. parvum*-associated complement activation may play an important role in the host defence against the tumour.

We thank Dr John Wisnant for the supply of *C. parvum* and Kathy Dandridge and Cecilia Kent for their excellent technical assistance.

Supported in part by USPH grant 1-501-RR05745-01, PHS contract No. 1-CB-33888 and by Burroughs Wellcome Company, Triangle Park, North Carolina. Drs Gutterman and Mavligit are the recipients of PHS career Development Awards No. 1-K04-CA-71007-01 and No. 1-K04-CA-00130 respectively.

### REFERENCES

ADLAM, C., BROUGHTON, E. S. & SCOTT, M. T. (1972) Enhanced Resistance of Mice to Infection with Bacteria Following Pretreatment with *C. Parvum*. *Nature, New Biol.*, **235**, 219.

BAND P. R. JAO-KING C. URTANSUN, R. & HARAPHONGSE, M. (1975) A Phase I Study of Intravenous *C. Parvum* in Solid Tumors. *Proc. Am. Ass. Cancer Res.*, **16**, 9.

COOPER, N. R. (1973) Activation of the Complement System. In *Contemporary Topics in Molecular Immunology*. Vol. 2. Ed. R. A. Reisfeld and W. J. Mandy. New York & London: Plenum Press.

DANNENBERG, A. M. (1975) Macrophages in Inflammation and Infection. *New Engl. J. Med.*, **293**, 489.

DAWES, J., TUACH, S. J. & MCBRIDE, W. H. (1974) Properties of an Antigenic Polysaccharide from *Corynebacterium parvum*. *J. Bacteriol.*, **120**, 24.

FEARON, D. T., RUDDY, S., SCHUR, P. H. & MCCABE, W. R. (1975) Properdin Pathway of Complement in Gram Negative Bacteremia. *New Engl. J. Med.*, **292**, 937.

GEWURTZ, H., SHIN. H. S. & MORGENHAGEN, S. E. (1968) Interaction of the Complement System with Endotoxic Lipopolysaccharide: Consumption of Each of the Six Terminal Complement Components. *J. exp. Med.*, **128**, 1049.

GHAFFAR, A., CULLEN, R. T. & WOODRUFF, M. F. A. (1975) Further Analysis of the Anti-tumour Effect *in vitro* of Peritoneal Exudate Cells from Mice Treated with *Corynebacterium parvum*. *Br. J. Cancer*, **31**, 15.

GÖTZE, O. & MÜLLER-EBERHARD, H. J. (1971) The C3 Activator System: An Alternate Pathway of Complement Activation. *J. exp. Med.*, **134**, Suppl. 90.

GUTTERMAN, J. U., MAVLIGIT, G. M., MCBRIDE, C. M., FREI, E. & HERSH, E. M. (1973) BCG Stimulation of Immune Responsiveness in Patients with Malignant Melanoma. *Cancer N.Y.* **32**, 321.

HALPERN B. N. BIOZZI G. STIFFEL, C. & MOUTON, D. (1966) Inhibition of Tumor Growth by Administration of Killed *Corynebacterium parvum*. *Nature, Lond.*, **212**, 853.

HALPERN, B., FRAY, A., CREPIN, Y., PLATICA, O. LORINET, A. M., RABOURDIN, A., SPARROS, L. & ISAC, R. (1973) *Corynebacterium parvum*, a Potent Immunostimulant in Experimental Infection and Malignancies. In *Immunopotentiation, Ciba Found. Symp.*, **18**, p. 217. Amsterdam: Associated Scientific Publishers.

HOWARD, J. G., BIOZZI, G., MOUTON, D. & LIACOPOULOS, P. (1967) An Analysis of the Inhibitory Effect of *C. Parvum* on Graft-*versus*-Host Disease. *Transplantation*, **5**, 1510.

HOWARD, J. G., SCOTT, M. T. & CHRISTIE, G. H. (1973) Cellular Mechanisms underlying the Adjuvant Activity of *C. Parvum*; Interactions of Activated Macrophages with T and B Lymphocytes. In *Immunopotentiation, Ciba Found. Symp.*, **18**, p. 101. Amsterdam: Associated Scientific Publishers.

IRIE, K., IRIE, R. F. & MORTON, D. L. (1975) Detection of Antibody and Complement Complexed *in vivo* on Membrane of Human Cancer Cells by Mixed Hemadsorption Technique. *Cancer Res.*, **35**, 1244.

ISRAEL, L., EDELSTEIN, R., DEPIERRE, A. & DIMITROV, N. (1975) Daily Intravenous Infusions of *C. parvum* in Twenty Patients with Disseminated Cancer: A Preliminary Report of Clinical and Biological Findings. *J. natn. Cancer Inst.*, **55**, 29.

JAFFE, E. S., SHEVACH, E. M., FRANK, M. M., BERARD, C. W. & GREEN, I. (1974) Nodular Lymphoma. Evidence for Origin from Follucular B Lymphocytes. *New Engl. J. Med.*, **290**, 813.

JAMES, K., CLUNIE, G. J. A., WOODRUFF, M. F. A., MCBRIDE, W. H., STIMSON, W. H., DREW, R. & CATTY, D. (1975) The Effect of *C. parvum* Therapy on Immunoglobulin Class and IgG Sub-class Levels in Cancer Patients. *Br. J. Cancer*, **32**, 310.

KLEIN, E., HALTERMANN, O. A., HELM, F., ROSNER, D., MILGRAM, H., ADLER, S., STOLL, H. L. JR., CASE, R. W., PRIOR, R. L. & MURPHY, G. P. (1975) Immunologic Approaches to the Management of Primary and Secondary Tumors Involving the Skin and Soft Tissues: Review of a Ten-year Program. *Transpl. Proc.*, **7**, 297.

LIKHITE, V. V. & HALPERN, B. N. (1974) Lasting Rejection of Mammary Adenocarcinoma Cell Tumor in DBA/2 Mice with Intratumor Injection of Killed *C. parvum. Cancer Res.*, **34**, 341.

MCBRIDE, W. H., WEIR, D. M., KAY, A. B., PEARCE, D. & CALDWELL, J. R. (1975) Activation of the Classical and Alternate Pathways of Complement by *C. parvum. Clin. exp. Immunol.*, **19**, 143.

MILAS, L., GUTTERMAN, J. U., BASIC, I., HUNTER, N., MAVLIGIT, G. M., HERSH, E. M. & WITHERS, H. R. (1974) Immunoprophylaxis and Immunotherapy for a Murine Fibrosarcoma with *C. granulosum* and *C. parvum. Int. J. Cancer*, **14**, 493.

MOAKE, J. L. & SCHULTZ, D. R. (1975) Hemolytic Anemia Associated with Multiple Antibodies and Low Serum Complement. *Am. J. Med.*, **58**, 431.

NISHIOKA, K. (1975) Complement System and Tumor Immunity. In *Host Defence against Cancer and its Potentiation*. Ed. D. Mizuno, *et al.* Baltimore, London, Tokyo: University Park Press.

OKUDA, T., YOSHIOKA, T., IKEKAWA, G., GHIHARA, G. & NISHIOKA, K. (1972) Anti-complementary Activity of Anti-tumor Polysaccharides. *Nature, New Biol.*, **238**, 59.

OLIVOTTO, M. & BOMFORD, R. (1974) *In Vitro* Inhibition of Tumour Cell Growth and DNA Synthesis by Peritoneal and Lung Macrophages from Mice Injected with *Corynebacterium parvum. Int. J. Cancer*, **13**, 478.

REED, R. C., GUTTERMAN, J. U., MAVLIGIT, G. M. & HERSH, E. M. (1975) Phase I Trial of Intravenous and Subcutaneous *C. parvum. Proc. Am. Soc. clin. Oncol.*, **16**, 9.

ROWLANDS, D. T. & DANIELE, R. P. (1975) Surface Receptors in the Immune Response. *New Engl. J. Med.*, **293**, 26.

RUDDY, S., GIGLI, I. & AUSTEN, K. F. (1972) The Complement System of Man (Four parts). *New Engl. J. Med.*, **287**, 489, 545, 592, 642.

SCHORLEMMER, H. U., DAVIES, P. & ALLISON, A. C. (1976) Ability of Activated Complement Components to Induce Lysosomal Enzyme Release from Macrophages. *Nature, Lond.*, **261**, 48.

VROON, D. H., SCHULTZ, D. R. & ZARCO, R. M. (1970) The Separation of Nine Components and Two Inactivators of Components of Complement in Human Serum. *Immuno-chemistry*, **7**, 43.

WAHL, S. M., IVERSON, G. M. & OPPENHEIM, J. J. (1974) Induction of Guinea Pig B-cell Lymphokine Synthesis by Mitogenic and Nonmitogenic Signals to Fc, Ig, and C3 Receptors. *J. exp. Med.*, **140**, 1631.

WANDS, J. R., MANN, E., ALPERT, E. & ISSELBACHER, K. J. (1975) The Pathogenesis of Arthritis Associated with Acute Hepatitis-B Surface Antigen-positive Hepatitis. Complement Activation and Characterization of Circulating Immune Complexes. *J. clin. Invest.*, **55**, 930.

WOLMARK, N. & FISHER, B. (1974) The Effect of a Single and Repeated Administration of *Corynebacterium parvum* on Bone Marrow Macrophage Colony Production in Syngeneic Tumor-bearing Mice. *Cancer Res.*, **34**, 2869.

WOODRUFF, M. F. A. & BOAK, J. L. (1966) Inhibitory Effect of Injection of *Corynebacterium parvum* on the Growth of Tumor Transplants in Isogeneic Hosts. *Br. J. Cancer*, **20**, 345.

WOODRUFF, M. F. A., MCBRIDE, W. H. & DUNBAR, W. (1974) Tumor Growth, Phagocytic Activity and Antibody Response in *C. parvum* Treated Mice. *Clin. exp. Immunol.*, **17**, 509.